# Supplementary material for: The interactome of intact mitochondria by cross-linking mass spectrometry provides evidence for coexisting respiratory supercomplexes
Source: Mol Cell Proteomics. 2017 Dec 8;17(2):216–32. doi: 10.1074/mcp.RA117.000470 (PMC5795388; doi:10.1074/mcp.RA117.000470)
Supplement: Supplemental Data [file supp_RA117.000470_133922_0_supp_23978_fzffwf.zip › spectra_annotation/mito_spectra_annotation/197-1-8-1-8-1.pdf]

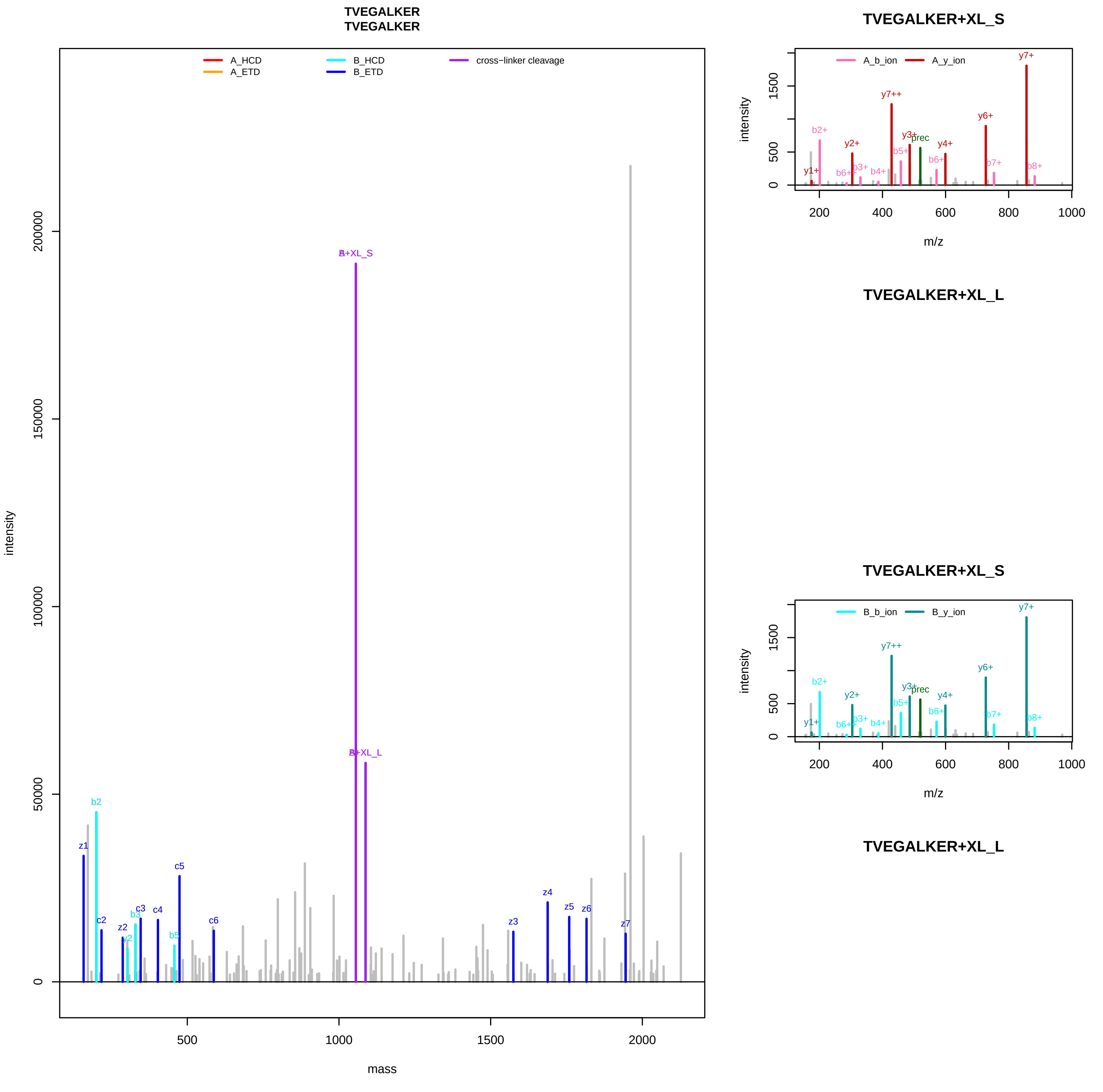
TVEGALKER
TVEGALKER
A_HCD
A_ETD
B_HCD
B_ETD
cross−linker cleavage
A+XL_S
B+XL_S
A+XL_L
B+XL_L
z1
b2
c2
z2
y2
b3
c3
c4
b5
c5
c6
z3
z4
z5
z6
z7
intensity
mass
0
50000
100000
150000
200000
500
1000
1500
2000
TVEGALKER+XL_S
A_b_ion
A_y_ion
y1+
b2+
y2+
b3+
b4+
b5+
b6+
y7++
y3+
prec
y4+
y6+
b7+
y7+
b8+
intensity
m/z
TVEGALKER+XL_L
TVEGALKER+XL_S
B_b_ion
B_y_ion
TVEGALKER+XL_L
